# Risk factor analysis and construction of prediction models for short-term postoperative complications in patients undergoing gastrointestinal tract surgery

**DOI:** 10.3389/fsurg.2022.1003525

**Published:** 2023-01-04

**Authors:** Hongming Cui, Dawei Zhao, Jingren Jian, Yifei Zhang, Mi Jian, Bin Yu, Jinchen Hu, Yanbao Li, Xiaoli Han, Lixin Jiang, Xixun Wang

**Affiliations:** ^1^ Department of Gastrointestinal Surgery, Yantai Yuhuangding Hospital, Qingdao University, Yantai, China; ^2^ Department of Surgical Department, Jinxiang Hongda Hospital Affiliated to Jining Medical University, Jining, China; ^3^ Department of Surgical Department, Yantai Yeda Hospital, Yantai, China

**Keywords:** gastrointestinal tumor, postoperative complications, stress response, fluid intake and output, clinicopathological characteristics, nomogram

## Abstract

**Purpose:**

To identify risk factors associated with short-term postoperative complications in patients with gastrointestinal cancer and develop and validate prediction models to predict the probability of complications.

**Methods:**

A total of 335 patients enrolled in the primary cohort of this study were divided into training and validation sets in a chronological order. Using univariate and multivariate logistic regression analyses, the risk factors for postoperative complications were determined, and nomogram prediction models were constructed. The performance of the nomogram was assessed with respect to the receiver operator characteristic and calibration curves.

**Results:**

Patients with complications had a stronger postoperative stress response and a longer duration of daily fluid intake/output ratio >1 after surgery. Logistic analysis revealed that body mass index (BMI), body temperature on POD4 (T.POD4), neutrophil percentage on POD4 (N.POD4), fasting blood glucose on POD4 (FBG.POD4), and the presence of fluid intake/output ratio <1 within POD4 were risk factors for POD7 complications, and that BMI, T.POD7, N.POD7, FBG.POD4, FBG.POD7, and the duration of daily fluid intake/output ratio >1 were risk factors for POD30 complications. The areas under the curve of Nomogram-A for POD7 complications were 0.867 and 0.833 and those of Nomogram-B for POD30 complications were 0.920 and 0.918 in the primary and validation cohorts, respectively. The calibration curves showed good consistency in both cohorts.

**Conclusion:**

This study presented two nomogram models to predict short-term postoperative complications in patients with gastrointestinal cancer. The results could help clinicians identify patients at high risk of complications within POD7 or POD30.

## Introduction

Gastric and colorectal cancers are among the top five causes of morbidity and mortality among all cancer patients in China (1, 2). Surgical treatment of gastrointestinal cancers remains an essential but aggressive treatment option (3). Meanwhile, postoperative complications represent the greatest obstacle that hinders recovery (4). When complications are treated inappropriately, they may lead to a rapid decline in the quality of life of the patient as well as an increase in medical expenses and mortality (5, 6). Therefore, early detection of and intervention for complications can effectively reduce the duration of hospitalization and medical expenditure.

Most gastrointestinal surgical complications are not obvious and are difficult to detect in the early stages (7). Therefore, early recognition and intervention are critical for postoperative treatment. Physical symptoms or laboratory tests can reveal early signs of postoperative complications; however, only a handful of studies have efficiently integrated these clinical data to assist in clinical decision-making (8).

The occurrence of complications is intimately linked to the stress response after surgery, and their occurrence usually indicates a high level of stress in the body. The high stress response in the early postoperative period also indicates that postoperative complications are likely to occur, which provides a reference point for predicting postoperative complications (9, 10).

Minimally invasive surgery combined with enhanced recovery after surgery is an important aspect of standardized gastrointestinal tumor management. Clinical trials have shown that they can improve short-term outcomes and long-term survival. However, postoperative complications remain an important clinical problem (11–13). The aim of the current study was to develop and validate a nomogram to estimate the possibility of postoperative complications in patients undergoing gastrointestinal tract surgery by incorporating routine indicators monitored in the postoperative setting.

## Materials and methods

### Patient selection

This study included patients with gastrointestinal tumors who underwent standard surgical treatment between June 2020 and July 2021 in the Gastrointestinal Unit of Yantai Yuhuangding Hospital, Qingdao University. The inclusion criteria were as follows: (i) age ≥18 years; (ii) no obvious contraindications in preoperative examination; (iii) preoperative pathology determined to be gastric or colorectal cancer; (iv) Eastern Cooperative Oncology Group performance status of ≤3; and (v) life expectancy of ≥6 months. The exclusion criteria were as follows: (i) without complete baseline examination; (ii) with the presence of secondary tumor or comorbidities on the preoperative examination that required emergency surgery; (iii) current or a history of malignancy in addition to gastrointestinal tumors; (iv) with other diseases that could either affect the study results or were uncontrollable.

This retrospective study was approved by the ethics committee of Yantai Yuhuangding Hospital, Qingdao University, and the requirement for informed consent was waived due to the retrospective nature of the study.

### Selection of research variables

Perioperative clinical data, such as baseline characteristics and laboratory results, were collected from each patient. The baseline characteristics included sex, age, body mass index (BMI), history of smoking and alcohol consumption, neoadjuvant chemoradiotherapy (nCR, and previous cardiovascular disease and diabetes. Laboratory data included preoperative white blood cell count, neutrophil percentage (N), fasting blood glucose (FBG), alanine aminotransferase, and aspartate aminotransferase levels on postoperative days 1 (POD1), 4 (POD4), and 7 (POD7). We also recorded the daily fluid intake and output per patient, including perioperative infusion, bleeding, drainage, and urine volume. We further calculated the daily fluid output and fluid difference using the following formula:

$$\begin{array}{rl} & \;\;Fluid\;\;output\;\left(\;ml\right)=VOL_{urine}+VOL_{drainage}+VOL_{invisible\;water\;loss}\\ & \;\;Fluid\;\;difference\;\left(\;ml\right)=VOL_{intake}-\;Fluid\;\;output\;\left(\;ml\right) \end{array}$$

Note that *VOL* is short for the letter “volume”. Invisible water loss was set at 900 ml and increased with increasing body temperature; by 200 ml when body temperature was between 37.3 °C and 37.7 °C, 500 ml between 37.8 °C and 38.3 °C, and 800 ml above 38.3 °C. Then, we assessed whether each patient had a fluid intake/output ratio <1 within POD4 (It means two consecutive days of daily fluid intake/output ratio <1 within POD4) and the final duration of daily fluid intake/output ratio >1. The first day of the first two consecutive days with a ratio <1 was estimated as the final duration of fluid intake/output ratio >1 from POD1. If there were two consecutive days without a ratio <1 within POD7, the duration was estimated as 7 days when there was a ratio <1 on POD7 and 8 days when there was still a ratio >1 on POD7. To explore the stress status of the patients, we measured levels of stress indicators, including perioperative C-reactive protein (CRP), interleukin-6 (IL-6), and cortisol.

In addition, according to each of their postoperative monitored vital signs, laboratory test results, and postoperative treatment measures, the presence of postoperative complications was assessed and recorded within POD7 or POD30 based on the first day of its appearance. Each postoperative complication was graded according to the Clavien–Dindo classification (14).

### Surgical treatment and postoperative management

Preoperative laboratory tests and examinations were performed in all patients to exclude clear contraindications to surgery. All surgeries were performed by five gastrointestinal specialists who performed more than 80 similar surgeries annually at Yantai Yuhuangding Hospital. At the end of the surgery, one or two drains were placed at the anastomosis and closed stump subcutaneously in all patients. All patients were routinely treated with prophylactic antibiotics, nutritional support, pain relief, and other symptomatic treatments after surgery. Postoperative routine blood tests and biochemistry were performed every 3 days. Gastrointestinal tract images was reviewed before discharge, and drains were removed if there were no signs of leakage. Simultaneously, patients were closely monitored for postoperative complications during the treatment process. Once they occurred, early intervention was provided.

### Development and validation of nomogram

We identified independent risk factors associated with postoperative complications by univariate and multifactorial logistic regression analyses, and the nomogram was built based on the independent risk factors in the multivariate analysis. First, we identified the independent risk factors associated with POD7 complications by using research variables and the presence or absence of a fluid intake/output ratio <1 within POD4 and then developed Nomogram A (Nomogram-A). Secondly, we identified the independent risk factors associated with POD30 complications by using research variables and the final duration of fluid intake/output ratio >1 within POD7 and then developed Nomogram B (Nomogram-B).

The recognition performance of Nomograms-A and -B were evaluated using receiver operator characteristic (ROC) curves in the training and validation sets (15). Comparisons between ROC curves were performed using the Delong test (16). The prediction accuracy of the nomogram was evaluated using calibration curves and the Hosmer–Lemeshow test (17).

### Statistical analysis

Statistical analyses were performed using SPSS (version 26.0) and R (version 3.6.2) software. Exact variables were analyzed using Student's t-test, and categorical variables were analyzed using the *χ*^2^ test or Fisher's exact test in the baseline table. Correlation analysis was performed using Spearman's correlation test. The independent risk factors were determined using univariate and multifactorial logistic regression analyses. Nomograms and calibration curves were plotted using the “RMS” software package. The ROC curves were plotted using the “pROC” software package. For all tests, a two-sided *P <* 0.05 was considered statistically significant.

## Results

### Patient characteristics

A total of 335 patients (225 men and 110 women) were included in this study and were divided chronologically into training (*n* = 223) and validation (*n* = 112) sets. The patients were also divided into a group with (*n* = 233) and without (*n* = 102) complications. Baseline patient characteristics and outcomes of the training and validation sets are shown in Table 1. There were no significant differences in the baseline patient characteristics between the training and validation sets, indicating good consistency between the two cohorts.

**Table 1:** Patient characteristics and short-term outcomes in the training and validation set.

Characteristics		Training set (*N* = 223)	Validation set (*N* = 112)	*P* value
Age (years, Mean ± SD)		65.83 ± 10.20	63.94 ± 11.11	0.121
Sex (%)	Male	146 (65.5)	78 (69.6)	0.444
	Female	77 (34.5)	34 (30.4)	
BMI (kg/m^2^, Mean ± SD)		24.45 ± 3.12	24.69 ± 3.16	0.500
Smoking, *n* (%)		75 (33.6)	45 (40.2)	0.238
Alcohol, *n* (%)		58 (26.0)	26 (23.2)	0.578
Cardiovascular disease, *n* (%)		78 (35.0)	35 (31.3)	0.496
Diabetes, *n* (%)		38 (17.0)	11 (9.8)	0.078
Surgical spot (%)	Stomach	94 (42.2)	52 (46.4)	0.457
	Intestines	129 (57.8)	60 (53.6)	
nCRT, *n* (%)		38 (17.0)	22 (19.6)	0.558
Fasting time (days, Mean ± SD)		5.3 ± 3.0	5.2 ± 2.1	0.685
First time of exhaust (days, Mean ± SD)		4.63 ± 2.40	4.62 ± 1.47	0.971
Time of urinary catheter withdrawal (days, Mean ± SD)		3.28 ± 2.69	3.26 ± 1.95	0.941
the duration of operation (minutes, Mean ± SD)		200.49 ± 56.73	208.30 ± 62.52	0.252
the duration of anesthesia (minutes, Mean ± SD)		243.81 ± 59.16	253.35 ± 65.34	0.180
Postoperative hospital stay (days, Mean ± SD)		8.7 ± 5.1	8.6 ± 4.2	0.825
Intraoperative blood transfusion		5	1	0.352
Maximum length of primary tumor				
(cm, Mean ± SD)		4.09 ± 2.24	4.15 ± 2.38	0.819
Lymph node metastasis, *n* (%)		92 (41.3)	47 (42.0)	0.901
Vascular invasion, *n* (%)		52 (23.3)	18 (16.1)	0.124
Nerve infiltration, *n* (%)		62 (27.8)	29 (25.9)	0.711

*BMI*, body mass index; *nCRT*, neoadjuvant chemoradiotherapy.

Among all patients, 146 had gastric cancer and 189 had colorectal cancer. Additionally, 60 patients had defined borderline resectable tumors, and 60 patients received 2–4 cycles of nCRT. The median and average postoperative hospital stay were 7 and 8.7 days, respectively.

In the group with complications, 86 and 102 patients developed postoperative complications within POD7 and POD30 (including those within POD7), respectively (Table 2). According to the Clavien–Dindo classification, 20 patients had major complications (Clavien–Dindo grades III/IV/V). One patient with Clavien–Dindo grade V was a man who suffered respiratory failure after radical gastric cancer surgery and died after ineffective treatment in the ICU. The complications in the remaining patients were effectively controlled or cured after standard treatment.

**Table 2:** Display of postoperative complications in the training and validation set.

		Training set (*N* = 223)	Validation set (*N* = 112)	*P* value
Complication	Total patients	68	34	0.960
	Anastomotic leakage	11	4	
	Abdominal hemorrhage	2	2	
	Gastrointestinal dysfunction^a^	7	3	
	Wound infection	4	4	
	Chylous leakage	3	/	
	Pleural effusion	6	3	
	Pneumonia	7	5	
	Respiratory and circulatory dysfunction^b^	9	2	
	Severe thrombosis^c^	3	/	
	Urinary abnormalities^d^	1	3	
	Metabolite or electrolyte imbalance^e^	8	3	
	Fever^f^	15	8	
Major complication^g^		13	7	0.818

We repeated the count if the patient had two or more comorbidities.

^a^ Postoperative gastroparesis, residual gastritis, intestinal obstruction, intestinal adhesions and recurrent diarrhea, etc.

^b^ Postoperative ventricular fibrillation, recurrent atrial fibrillation, heart failure, respiratory failure and unexplained severe chest tightness, etc.

^c^ Thrombotic pulmonary embolism and cerebral infarction, severe venous thrombosis, etc.

^d^ Urinary tract infection, hematuria, urethral fistula, etc.

^e^ Persistent hypokalemia or hyperglycemia, abnormal liver and kidney function, etc.

^f^ Transient temperature above 38.5 °Cor temperature above 37.5 °C for 2 or more days.

^g^ Clavien-dindo Grade III/IV/V.

### Study of the correlation between stress and postoperative complications

We monitored the preoperative CRP, IL-6, and cortisol levels on POD1, POD4, and POD7 in partial patients from the training set (*n* = 168). Comparing the stress indicators between the groups with and without complications, the CRP, IL-6, and cortisol levels were significantly higher in the group with complications than in those without complications on POD1, POD4, and POD7 (*P <* 0.05); however, there was no significant difference between the groups before surgery (*P >* 0.05) (Table 3).

**Table 3:** Perioperative fluid volume in patients with or without complications.

		With complications (*N* = 102)	Without complications (*N* = 233)	*P* value
Fluid difference (mL, Mean ± SD)	POD1	402.09 ± 626.50	350.36 ± 754.66	0.100
	POD2	304.39 ± 617.95	98.29 ± 630.61	0.006
	POD3	301.04 ± 626.51	124.17 ± 649.42	0.021
	POD4	72.90 ± 670.03	−339.15 ± 731.84	<0.001
	POD5	−68.50 ± 769.37	−553.18 ± 752.41	<0.001
	POD6	−205.14 ± 879.00	−735.72 ± 741.77	<0.001
	POD7	−530.46 ± 608.57	−685.11 ± 732.79	0.035
Intraoperative fluid intake (mL, Mean ± SD)		1827.27 ± 702.56	1736.08 ± 600.45	0.303
Intraoperative bleeding volume (mL, Mean ± SD)		70.29 ± 201.33	47.52 ± 78.88	0.271
Presence of ratio <1 (%)		9 (10.5)	91 (36.5)	<0.001
Duration of ratio >1 (days, Mean ± SD)		6.12 ± 1.59	3.86 ± 1.76	<0.001

*Fluid difference* = daily fluid intake—output; *Presence of ratio <1*, The presence of fluid intake/output ratio < 1 within POD4 was analyzed with the occurrence of POD7 complications; *Duration of ratio > 1*, the final duration of fluid intake/output >1.

The results of this study further confirm that patients with postoperative complications generally have a stronger postoperative stress response than those without postoperative complications, and the presence of a strong stress response in the early postoperative period may reflect the occurrence of postoperative complications.

### Study of the correlation between fluid volume and postoperative complications

This study also analyzed perioperative fluid volumes between the groups of patients with and without postoperative complications. Intraoperative bleeding and infusion volumes in the group with complications were not significantly different from those in the group without complications (*P >* 0.05) (Table 4). In contrast, during postoperative fluid therapy, the daily fluid difference from POD2 in the group with complications was lower than that in the group without complications (*P <* 0.05), and the fluid gap increased significantly from POD4. This gap between the two groups persisted until the last day of statistical analysis. Short-term postoperative complications tended to occur 3–5 days after surgery; therefore, we considered this increase in variance to be related to the timing of postoperative complications. We also compared the presence or absence of fluid intake/output ratio <1 within POD4 and the final duration of fluid intake/output ratio >1 between both groups. Patients with complications were less likely to have a fluid intake/output ratio <1 within POD4 (10.5% vs. 36.5%, *P <* 0.05) and had a significantly longer duration of fluid intake/output ratio >1 postoperatively than those without complications (6.18 days vs. 3.86 days, *P <* 0.05).

**Table 4:** Perioperative stress index in patients with or without complications.

	With complications	Without complications	*P* value
CRP.pre (mg/L, Mean ± SD)	5.98 ± 8.85	3.80 ± 5.94	0.488
CRP.POD1 (mg/L, Mean ± SD)	63.22 ± 30.72	38.88 ± 23.89	0.002
CRP.POD4 (mg/L, Mean ± SD)	106.35 ± 53.65	31.37 ± 15.64	<0.001
CRP.POD7 (mg/L, Mean ± SD)	81.86 ± 60.15	29.36 ± 30.89	<0.001
IL-6.pre (U/ml, Mean ± SD)	3.11 ± 3.29	11.92 ± 31.40	0.412
IL-6.POD1 (U/ml, Mean ± SD)	160.86 ± 115.78	92.16 ± 95.99	0.023
IL-6.POD4 (U/ml, Mean ± SD)	114.65 ± 52.72	26.04 ± 22.68	<0.001
IL-6.POD7 (U/ml, Mean ± SD)	79.11 ± 78.25	19.00 ± 14.76	0.013
Cortisol.pre (nmol/L, Mean ± SD)	325.14 ± 88.16	344.23 ± 93.43	0.598
Cortisol.POD1 (nmol/L, Mean ± SD)	582.17 ± 137.51	413.83 ± 151.13	<0.001
Cortisol.POD4 (nmol/L, Mean ± SD)	663.18 ± 110.77	390.39 ± 136.35	<0.001
Cortisol.POD7 (nmol/L, Mean ± SD)	576.66 ± 156.07	365.34 ± 115.12	<0.001

*CRP.pre*, preoperative C-reactive protein; *CRP.POD1*, CRP on the postoperative day 1; *CRP.POD4*, CRP on the postoperative day 4; *CRP.POD7*, CRP on the postoperative day 7; *IL-6*, interleukin-6; IL-6 and Cortisol in the same way.

Through our study, we considered that changes in postoperative fluid difference may be associated with the development of postoperative complications and further discuss whether they could be risk factors for the development of postoperative complications.

### Development and validation of nomogram

**Figure 1:**
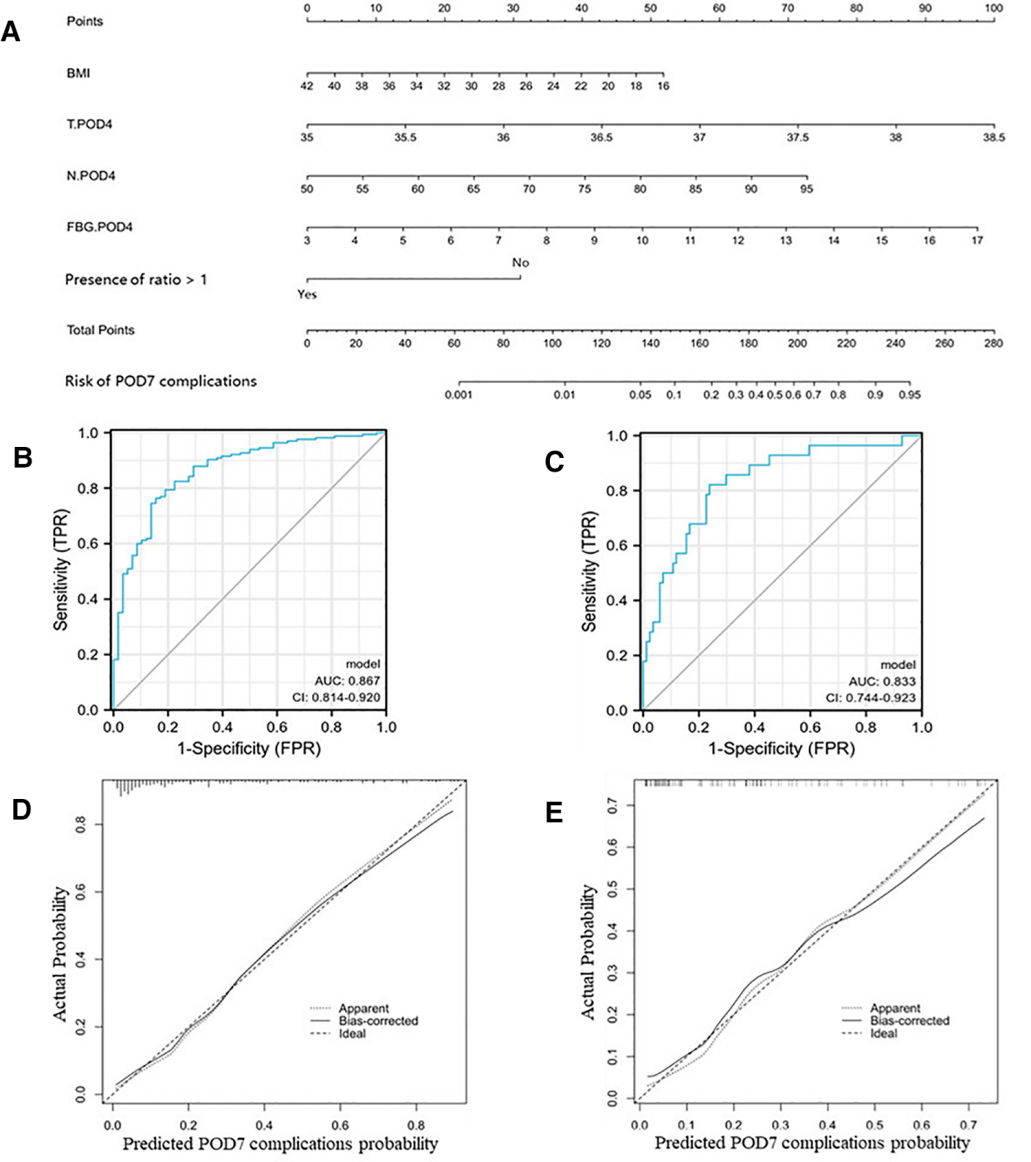
Nomogram-A and performance of the nomogram. (**A**) The possibility of POD7 complication was estimated by summing the scores corresponding to each risk factor. ROC and calibration curves of the nomogram for the probability of POD7 complication in the training set (**B**,**D**) and the validation set (**C,E**). In the calibration curve, the *y*-axis represents the probability of actual POD7 complication occurring and the *x*-axis represents the predicted probability. The wide dashed line represented a perfect prediction of the ideal model, and the solid line represented the actual performance of the Nomogram-A. The closer they were, the better the prediction performed.

First, we used clinical data within POD4 and identified five risk factors associated with the occurrence of complications within POD7 by univariate and multifactorial logistic regression analyses (Table 5): BMI, body temperature on POD4 (T.POD4), neutrophil percentage on POD4 (N.POD4), FBG on POD4 (FBG.POD4), and fluid intake/output ratio <1 within POD4. Subsequently, we developed Nomogram-A (Figure 1A). In the training set, the nomogram yielded an area under the curve (AUC) of 0.867 (95% CI, 0.814–0.920) with a sensitivity of 0.764 and a specificity of 0.845 (Figure 1B). In the validation set, the nomogram exhibited an AUC of 0.833 (95% CI, 0.744–0.923) with a sensitivity of 0.821 and a specificity of 0.762 (Figure 1C).

**Table 5:** Univariate and multivariate analysis for POD7 complication in the training set.

Characteristics	Univariate analysis		Multivariate analysis	
	OR (95%Cl)	*P* value	OR (95%Cl)	*P* value
Cardiovascular disease	3.52 (1.89–6.56)	<0.001		
Diabetes	5.69 (2.72–11.93)	<0.001		
Age ≥ 65	2.89 (1.45–5.75)	0.003		
BMI ≤ 25	2.96 (1.51–5.81)	0.002	2.60 (1.02–6.62)	0.045
T.POD4 ≥ 37	9.24 (3.13–27.31)	<0.001	6.30 (1.43–27.73)	0.015
WBC.POD4 ≥ 9.5	3.89 (1.92–7.87)	<0.001		
N.POD4 ≥ 75	6.83 (3.44–13.55)	<0.001	3.60 (1.46–8.87)	0.005
FBG.POD4 ≥ 6.2	12.92 (6.13–27.23)	<0.001	8.28 (3.20–21.42)	<0.001
Absence of ratio <1	5.62 (2.29–13.89)	<0.001	5.56 (1.63–18.87)	0.006

*BMI*, body mass index, kg/m^2^; *T.POD4*, the temperature on the postoperative day 4, °C; *WBC.POD4*, white cell count on the postoperative day 4, ×10^9^/l; *N.POD4*, neutrophil percentage on the postoperative day 4, %; *FBG.POD4*, fasting blood glucose on the postoperative day 4, mmol/l; *Absence of ratio <1*, the absence of fluid intake/output <1 within POD4.

**Figure 2:**
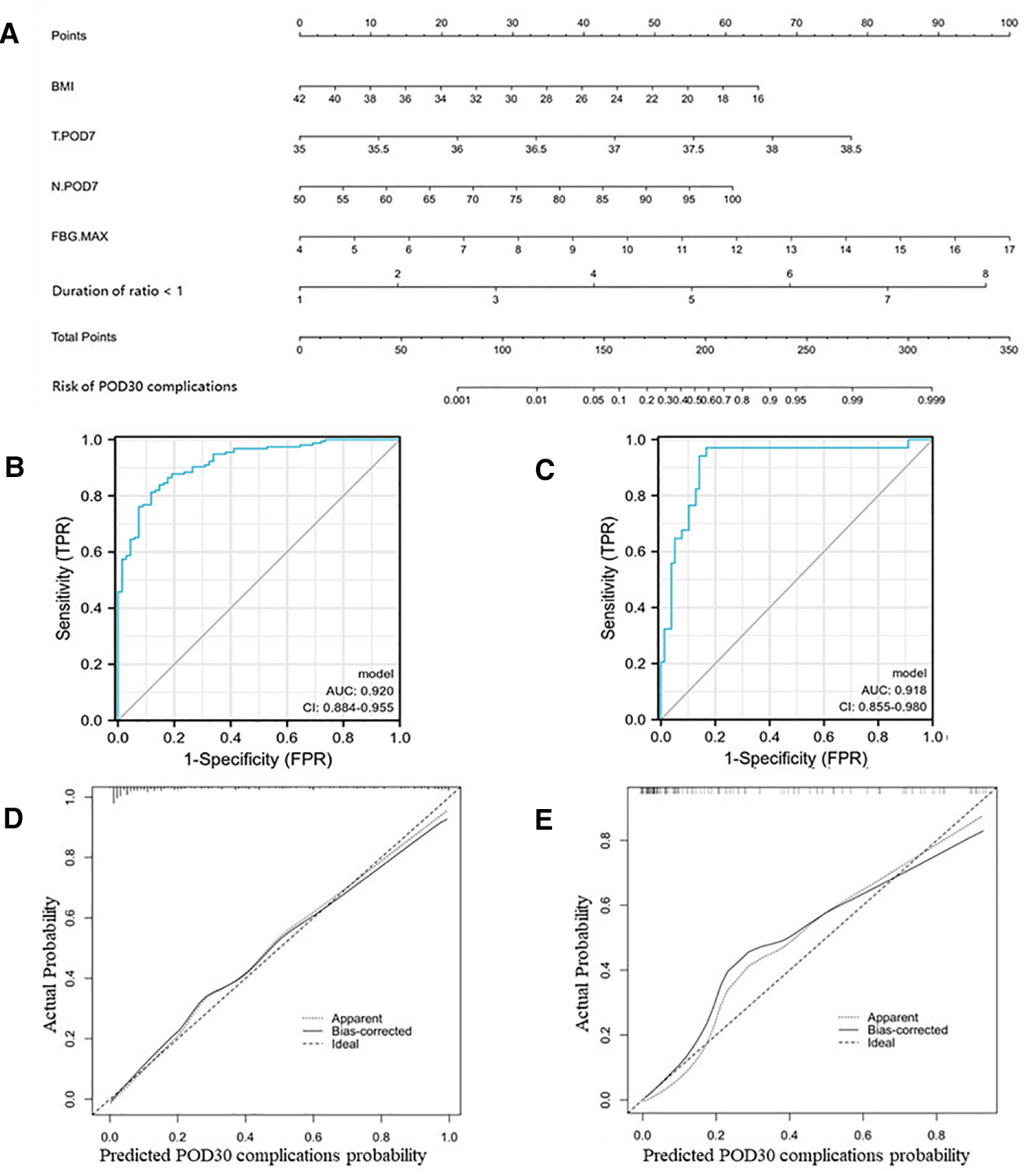
Nomogram-B and performance of the nomogram. (**A**) The possibility of POD30 complication was estimated by summing the scores corresponding to each risk factor. ROC and calibration curves of the nomogram for the probability of POD30 complication in the training set (**B,D**) and the validation set (**C,E**). In the calibration curve, the *y*-axis represents the probability of actual POD30 complication occurring and the *x*-axis represents the predicted probability. The wide dashed line represented a perfect prediction of the ideal model, and the solid line represented the actual performance of the Nomogram-B. The closer they were, the better the prediction performed.

Similarly, we identified six independent risk factors associated with the occurrence of complications within POD30 in Table 6, including BMI, body temperature on POD7 (T.POD7), neutrophil percentage on POD7 (N.POD7), FBG on POD4 and POD7 (FBG.POD4 and FBG.POD7), and duration of fluid intake/output ratio >1. To avoid data redundancy, we compared FBG.POD4 and FBG.POD7 and further defined the higher values as the maximum FBG (FBG.MAX). Further, we used BMI, T.POD7, N.POD7, FBG.MAX, and a fluid intake/output ratio of >1 to develop Nomogram-B (Figure 2A). In the training set, the nomogram yielded an AUC of 0.920 (95% CI, 0.884–0.955) with a sensitivity of 0.813 and specificity of 0.882 (Figure 2B). In the validation set, the nomogram exhibited an AUC of 0.918 (95% CI, 0.855–0.980) with a sensitivity of 0.971 and a specificity of 0.833 (Figure 2C).

**Table 6:** Univariate and multivariate analysis for POD30 complication in the training set.

Characteristics	Univariate analysis		Multivariate analysis	
	OR (95%Cl)	*P* value	OR (95%Cl)	*P* value
Cardiovascular disease	3.03 (1.67–5.48)	<0.001		
Diabetes	5.49 (2.62–11.53)	<0.001		
Age ≥ 65	2.54 (1.35–4.78)	0.004		
BMI ≤ 25	3.29 (1.73–6.26)	<0.001	3.62 (1.12–11.64)	0.031
T.POD4 ≥ 37	14.34 (3.99–51.49)	<0.001		
T.POD7 ≥ 37	7.73 (2.02–29.54)	0.003	7.87 (1.05–59.20)	0.045
WBC.POD4 ≥ 9.5	5.38 (2.64–10.96)	<0.001		
WBC.POD7 ≥ 9.5	7.25 (3.11–16.91)	<0.001		
N.POD4 ≥ 75	7.33 (3.83–14.04)	<0.001		
N.POD7 ≥ 75	12.55 (6.13–25.72)	<0.001	6.08 (1.82–20.27)	0.003
FBG.POD4 ≥ 6.2	12.11 (6.10–24.05)	<0.001	6.62 (1.86–23.53)	0.003
FBG.POD7 ≥ 6.2	12.48 (6.32–24.67)	<0.001	5.02 (1.43–17.68)	0.012
(Duration of ratio >1) ≥ 6	10.87 (5.60–21.13)	<0.001	8.19 (2.83–23.70)	<0.001

*BMI*, body mass index, kg/m^2^; *T.POD4*, the temperature on the postoperative day 4, °C; T.PO*D7*, the temperature on the postoperative day 7, °C; *WBC.POD4*, white cell count on the postoperative day 4, ×10^9^/l; *WBC.POD7*, white cell count on the postoperative day 7, ×10^9^/l; *N.POD4*, neutrophil percentage on the postoperative day 4, %; *N.POD7*, neutrophil percentage on the postoperative day 7, %; *FBG.POD4*, fasting blood glucose on the postoperative day 4, mmol/l; *FBG.POD7*, fasting blood glucose on the postoperative day 7, mmol/l; *Duration of ratio > 1*, the final duration of fluid intake/output > 1.

The calibration curves and Hosmer–Lemeshow test showed good agreement between the predicted and actual probabilities of the two nomograms in the training and validation sets (*P_A_*_ _= 0.755 vs. 0.738 (Figures 1D,E), *P_B_*_ _= 0.768 vs. 0.125 (Figures 2D,E)).

## Discussion

In the present study, we successfully developed and validated nomograms for predicting postoperative complications within POD7 and POD30 in patients with gastrointestinal tumors. Both nomogram models were developed based on routine clinical indicators, including body temperature, neutrophil percentage, fasting glucose, and fluid volume within POD7. The AUCs of Nomogram-A and Nomogram-B were both greater than 0.8 in the training and validation sets, which may achieve the best performance in predicting the likelihood of postoperative complications. Comprehensively, this model is used to identify people at high risk of postoperative complications, thereby reducing its impact on postoperative recovery. The goal of both models is to improve short-term outcomes and quality of life after surgery and to reduce healthcare costs.

It has been documented that the occurrence of postoperative complications is closely associated with stress, and their occurrence usually indicates a strong stress response in the patient that induces hypermetabolism (9, 18). The main reparative cells and leukocytes increase in activity and number to satisfy the glucose supply for body recovery during this period (19). Meanwhile, with the upregulation of pro-inflammatory cytokines and acute-phase proteins and activation of the hypothalamic-pituitary-adrenal axis, there is an increase or decrease in the levels of some hormones, including cortisol, catecholamines, insulin, and glucagon, which promote glycogen catabolism and gluconeogenesis in muscle tissue that induces hyperglycemia to compensate for the “concentration gradient” needed for tissue repair (20). In addition, with the influence of hormones, capillary permeability and urine concentration increase (21). A large amount of fluid is stored in the interstitial space, resulting in tissue edema and is known as “water and sodium retention” When the stress response is reduced due to intervention and self-healing, excessive fluid is reabsorbed into the blood and excreted in the urine (22).

In our study, we also found a higher level of the stress response in patients with postoperative complications. In line with the study by Espiner EA, the alteration of stress results in increased water and sodium retention and further leads to more liquid intake than output, ultimately resulting in a higher daily fluid intake/output ratio (23). And we further observed that the presence of fluid intake/output ratio <1 within POD4 was related to the occurrence of complications within POD7, and that patients who developed complications within POD30 had a longer duration of fluid intake/output ratio >1. Under theoretical frameworks of perioperative stress response, we investigated the relationship between postoperative complications and fluid intake and output. The results demonstrated that the fluid intake/output ratio could be a convincing risk factor and predictor for the occurrence of complications.

Our study also validated the correlation between postoperative complications and routine monitoring indicators, such as body temperature, neutrophil percentage, and FBG (24). Slight changes in one or more these indicators were often overlooked by clinicians, leading to a failure of early identification of complications. Numerous studies have shown that changes in routine monitoring indicators may be affected by the regulation of inflammatory cytokines and hormones during the hypermetabolic period of the stress response (25, 26). In our analysis, stress level was positively correlated with certain routine monitoring indicators, such as body temperature, neutrophil percentage and FBG levels. Considering the correlation between acute stress pathophysiological and postoperative complications, these routine monitoring indicators may be potential risk factors and candidate predictors for postoperative complications (27–30). In addition, it is generally believed that perioperative routine monitoring indicators also reflect inflammation level. Elevated level of these indicators indicated a higher inflammatory level in patients with complications (31). In this study, we use acute stress-associated indicators to reflect inflammation level, thereby provide an early warning tool for complications. For patients with gastrointestinal tumor undergoing radical surgery, surgery- and tumor-associated factors, such as loss of blood, tissue injury, infection, impaired nutritional status and immunocompromised status, may place the patients in a high risk of complications (10, 32, 33). Timely identification of postoperative complication will urge us to recognize the problems in patient management, and timely intervention may reduce likelihood of severe advent events (33, 34). Mechanically, timely intervention for complication will reduce overconsumption of protein and duration of immune dysregulation, and further decrease the possibility of malnutrition, infection, thrombosis, etc. The final objective is to improve outcomes for patients and save medical resources (35, 36). Similarly, evidence by previous researches showed that preoperative glucocorticoid therapy combined with fast-track surgery can attenuate the inflammatory response, avoid organ dysfunction, and prevent the occurrence of complications, resulting in improved short-term outcome (37–39).

Our study had some limitations. First, this study was a retrospective study that might have led to bias. Further prospective data are needed to validate the accuracy of our model. Second, a small number of patients were not included in this study owing to missing data, which resulted in a higher population of the group with complications. This may also lead to selection bias. Third, because of the high cost of measuring stress indicators, we only collected partial data from training set. And we will further consider ending the limitations by future prospective studies needed.

## Conclusion

This study presents two nomogram models for predicting short-term postoperative complications in patients with gastrointestinal tumors. Our results can help clinicians identify patients at high risk of complications within POD7 or POD30. In addition, we explored the correlation between postoperative complications and fluid intake and output under the framework of pathophysiology and stress response and successfully identified new risk factors for complications. This study also provides a novel idea for predicting postoperative complications.

## Data Availability

The raw data supporting the conclusions of this article will be made available by the authors, without undue reservation.
